# Propofol-induced anesthesia involves the direct inhibition of glutamatergic neurons in the lateral hypothalamus

**DOI:** 10.3389/fnins.2024.1327293

**Published:** 2024-01-12

**Authors:** Yan Huang, Yong Xiao, Linji Li, Xinglong Feng, Weixing Ding, Feng Cai

**Affiliations:** ^1^Department of Anesthesiology, Nanchong Central Hospital, Second Clinical Medical College of North Sichuan Medical College, Nanchong, China; ^2^Emergency Department of the General Hospital of the Tibet Military Region, Lhasa, China; ^3^Qujing Secend Peopie’s Hospital, Department of Pain, Qujing, Yunnan, China; ^4^Department of Urologyand Neurocardiothoracic Surgery, 927 Hospital of the Joint Logistics Support Force of the Chinese People’s LiberationArmy, Puer, China

**Keywords:** anesthesia, propofol, lateral hypothalamus, glutamatergic neurons, hM4Di receptors

## Abstract

Propofol is the most widely used intravenous general anesthetic; however, the neuronal circuits that mediate its anesthetic effects are still poorly understood. Glutamatergic neurons in the lateral hypothalamus have been reported to be involved in maintenance of arousal and consciousness. Using Vglut2-Cre transgenic mice, we recorded this group of cells specifically and found that propofol can directly inhibit the glutamatergic neurons, and enhance inhibitory synaptic inputs on these cells, thereby reducing neuronal excitability. Through chemogenetic interventions, we found that inhibition of these neurons increased the duration of propofol-induced anesthesia and reduced movement in the animals after the recovery of right reflex. In contrast, activating this group of cells reduced the duration of propofol anesthesia and increased the animals’ locomotor activity after the recovery of right reflex. These results suggest that propofol-induced anesthesia involves the inhibition of glutamatergic neurons in the lateral hypothalamus.

## Introduction

Intravenous general anesthetics are of great significance to modern surgical operation. Propofol is the most widely used intravenous anesthetic and is characterized by its favorable recovery profile (Choi et al., 2017; Mehta et al., 2017). A great deal of effort has gone into dissecting the neural circuitry and molecular targets underlying the effects of intravenous general anesthetics including propofol. Many studies have reported that propofol functions directly through generating a wide range of inhibitory impacts on neocortical regions, such as frontal cortex and entorhinal cortex (Li et al., 2016; Kobayashi and Oi, 2017; Luo et al., 2019). It is widely considered that these inhibitory effects might be attributed to that propofol could activate γ-aminobutyric acid type A receptors (GABA_*A*_Rs) and thus increase the inhibitory inputs (Reine et al., 1992; Chen et al., 1999; Jeong et al., 2011). Despite the pioneering work, the neuronal circuits and molecular targets that mediate anesthetic effects of propofol are still not completely understood. A systematic understanding of the anesthetic mechanism of propofol will contribute to the development of new anesthetics.

It has been reported that the cortical activities are tightly regulated by multiple subcortical arousal- and sleep-promoting brain regions. In fact, general anesthetics may act either by inhibiting subcortical arousal- promoting systems or activating sleep-promoting systems in the brain. For example, sevoflurane could inhibit the wakefulness-promoting neurons, such as dopamine D1 receptor-positive neurons in the nucleus accumbens (Bao et al., 2021), hypocretinergic neurons in the lateral hypothalamus (LH) (Kelz et al., 2008) and medial parabrachial neurons (Xu et al., 2020). Additionally, general anesthetics including dexmedetomidine, isoflurane, ketamine and propofol also activate sleep-promoting neurons such as the supraoptic nucleus to contribute to their anesthetic action (Jiang-Xie et al., 2019; Hua et al., 2020).

It should be noted that glutamatergic neurons in the LH have been reported to be involved in maintenance of arousal. Chemogenetic activation of LH glutamatergic neurons induced an increase in arousal that lasted for 6 h. In contrast, suppression of LH glutamatergic neuronal activity caused a reduction in wakefulness (Wang et al., 2021). Although the crucial role of the glutamatergic neurons in the LH in the maintenance of wakefulness has been established, it is still unclear whether propofol affects the activity of these neurons. Here, we have discovered a direct inhibition of glutamatergic neurons in the LH by propofol, which is involved in its anesthetic action. These results unveil a novel neural mechanism underlying the anesthetic effects of propofol and provide the targets for research and development of new anesthetic drugs.

## Results

### Propofol generates hyperpolarization of the wakefulness-related glutamatergic neurons in the LH

**FIGURE 1:**
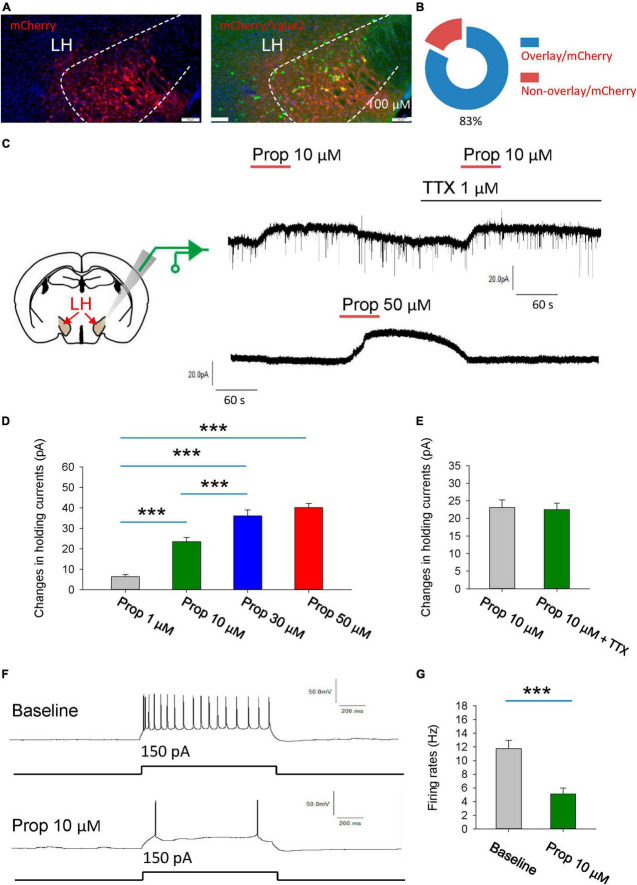
Propofol induces hyperpolarization of the glutamatergic neurons in the LH. **(A,B)** Fluorescence *in situ* hybridization (FISH) to detect the vesicular glutamate transporter 2 (VGLUT2, encoded by *Slc17a6*), noting that mCherry is selectively expressed in *Slc17a6*-positive neurons. **(C)** Schematic diagram for recording glutamatergic neurons in the LH (Left). Example electrophysiological traces before, during and after bath application of the 10 and 50 μM propofol (Right). **(D)** Effects of propofol on the holding currents of glutamatergic neurons in the LH. **(E)** Effects of propofol on the holding currents in the presence of TTX (1 μM) to block action potentials-dependent synaptic transmissions. **(F,G)** Effects of propofol on the firing rates of glutamatergic neurons in response to inward current stimuli. ***P < 0.001.

To visualize glutamatergic neurons in the LH, AAV2/9-EF1α-DIO- mCherry was injected into the LH of vGlut2-Cre mice to label the glutamatergic neurons with the mCherry. To further validate the specificity of the mCherry expression, we used fluorescence *in situ* hybridization (FISH) to detect the vesicular glutamate transporter 2 (VGLUT2, encoded by *Slc17a6*), and found that mCherry is selectively expressed in *Slc17a6*-positive neurons (Figures 1A, B).

We initially explored the effect of the propofol on the glutamatergic neurons in the LH (Figure 1C). The glutamatergic neurons were specifically recorded from the brain slices. The voltage-clamp recordings were performed to test the effects of propofol on the holding currents of glutamatergic neurons. Bath application (1–50 μM, 1 min) of propofol induced outward currents in the recorded glutamatergic neurons. The amplitude of outward currents induced by propofol was concentration-dependent (Figures 1C, D).

Next, we investigated whether propofol directly affects membrane intrinsic properties. To test this, the hold currents were recorded in the presence of tetrodotoxin (TTX, 1 μM) to block action potentials-dependent synaptic transmissions. Under this condition, we found that propofol still induced outward currents in the glutamatergic neurons (Figure 1E), suggesting that propofol directly influences the membrane properties of the glutamatergic neurons.

Additionally, propofol (10 μM) significantly reduced the firing rates of these neurons in response to inward current stimuli (Figures 1F, G). In sum, these data suggest that propofol suppresses the excitability of the glutamatergic neurons in the LH.

### Propofol enhances the GABAergic inhibitory inputs on glutamatergic neurons

**FIGURE 2:**
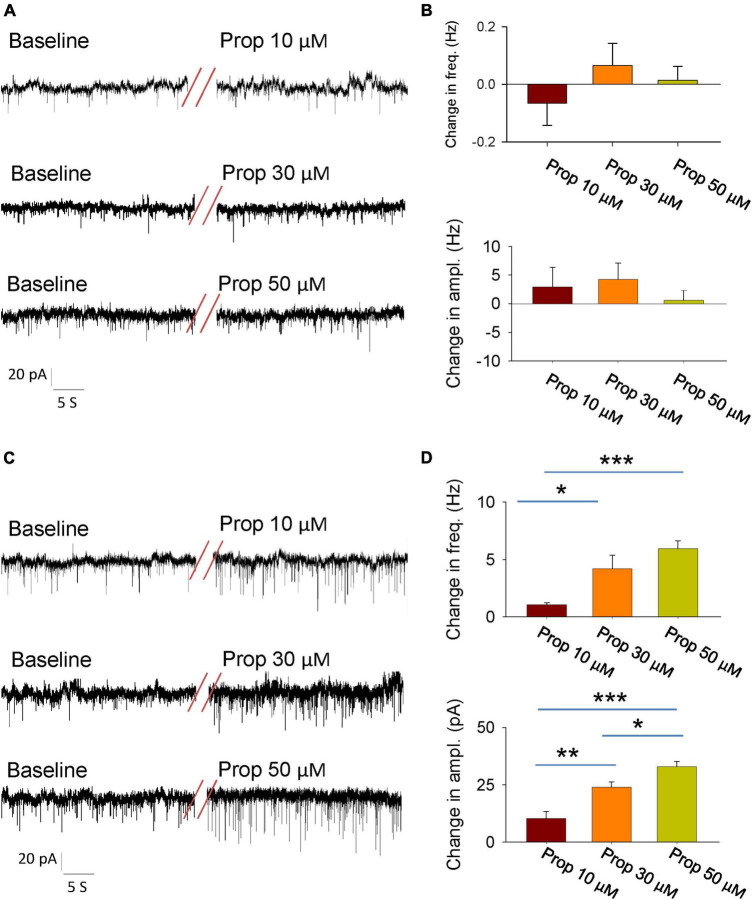
Effects of propofol synaptic transmissions of the glutamatergic neurons in the LH. **(A)** An example electrophysiological trace showing the SEPSCs of the glutamatergic neurons before and during bath application of the propofol. **(B)** Effects of propofol (10, 30, and 50 μM) on the frequency (top) and amplitude (down) of the SEPSCs. **(C)** An example electrophysiological trace showing the SIPSCs the of glutamatergic neurons before and during bath application of the propofol. **(D)** Effects of propofol (10, 30, and 50 μM) on the frequency (top) and amplitude (down) of the SIPSCs. *,**,***P < 0.001.

The activity of individual neurons as well as neural network is tightly controlled by the synaptic transmissions. Thus, we next explored whether propofol influences the synaptic transmissions of the glutamatergic neutrons in the LH. We firstly recorded the spontaneous excitatory postsynaptic currents (SEPSCs) in the presence of GABA_*A*_ receptor antagonist bicuculline. The glutamatergic neurons of the LH exhibited a continuous level of fast excitatory synaptic events. Bath application of propofol did not affect the amplitude and frequency of SEPSCs in these glutamatergic neurons (Figures 2A, B).

To test the regulation of GABA_*A*_ receptor-mediated spontaneous inhibitory postsynaptic currents (SIPSCs) by propofol, we blocked the ionotropic glutamate receptors by using 6-cyano-7-nitroquinoxaline-2, 3-dione (CNQX) and DL-2-amino-5-phosphonopentanoic acid (AP5). After recording stable baseline of SIPSCs, bath application of propofol remarkably increased the frequency and amplitude of these currents (Figures 2C, D). These results indicate that propofol increases inhibitory inputs of the glutamatergic neurons without affecting their excitatory inputs.

### Chemogenetic inhibition of glutamatergic neurons in the LH facilitates induction of and prolongs emergence from propofol anesthesia

**FIGURE 3:**
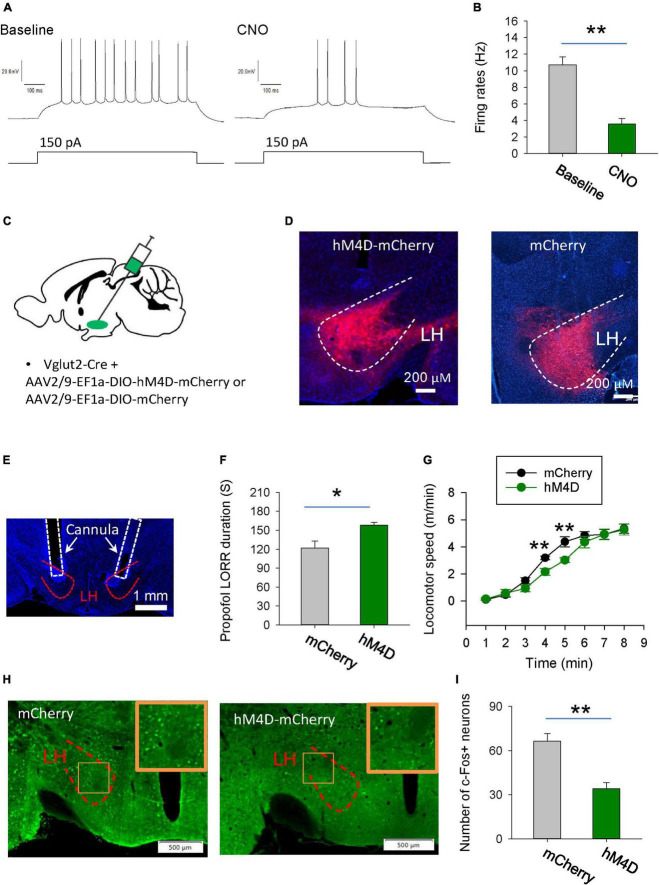
Chemogenetic inhibition of glutamatergic neurons in the LH facilitates induction of propofol anesthesia. **(A,B)** Effect of CNO on the firing rates of the hM4D-mCherry-positive neurons in the LH. **(C)** Bilateral injection of viruses into the LH region of vglut2-Cre mice. **(D)** Expression of the hM4D-mCherry and mCherry was restricted to cells in the LH. **(E)** A representative image showing the cannula implanted in the LH. **(F)** Chemogenetic inhibition of glutamatergic neurons in the LH decreased the duration of propofol (10 mg/kg, i.v.)-induced loss of LORR. **(G)** Effect of chemogenetic inhibition of glutamatergic neurons on locomotion of the mice following recovery of the LORR. **(H)** Representative images showing the c-Fos expression in the LH after application of CNO in the mCherry and hM4D-mCherry groups. The inset is an enlarged view of c-Fos expression in the LH. **(I)** Effect of chemogenetic inhibition on the c-Fos expression in the LH. *,**P < 0.001.

To investigate the behavioral consequences of chemogenetic inhibition of glutamatergic neurons in the LH, AAV-DIO-hM4Di-mCherry was injected into the LH of vGlut2-Cre mice to inhibit glutamatergic neurons. Whole-cell recordings of hM4Di-mCherry-positive glutamatergic neurons from acute brain slices showed a decreased number of action potentials with bath-applied clozapine N-oxide (CNO), implying that CNO was sufficient to inhibit hM4Di-mCherry-positive neurons in the LH (Figures 3A, B). The hM4Di-mCherry and mCherry were restrictedly expressed in the LH (Figures 3C, D). Compared with those in the vehicle group, chemogenetic inhibition of glutamatergic neurons in the LH decreased the duration of propofol (10 mg/kg, i.v.)-induced loss of right reflex (LORR) (Figures 3E, F). Even after they recovered from LORR, the locomotion of the mice with CNO injection was impaired for several minutes following propofol injection compared with control group (Figure 3G). These results suggest that glutamatergic neurons in the LH are involved in the propofol anesthesia.

We further detected the changes in neuronal activity in the LH region by c-Fos staining. Compared with the mCherry group, when LH glutamatergic neurons were inhibited by CNO during anesthesia, c-Fos positive neurons were significantly reduced (Figures 3H, I). These results further suggest that chemogenetic intervention affecting propofol anesthesia may indeed be inhibiting the neural activity in the LH region.

### Activation of the glutamatergic neurons in the LH delays induction of and accelerates emergence from propofol anesthesia

**FIGURE 4:**
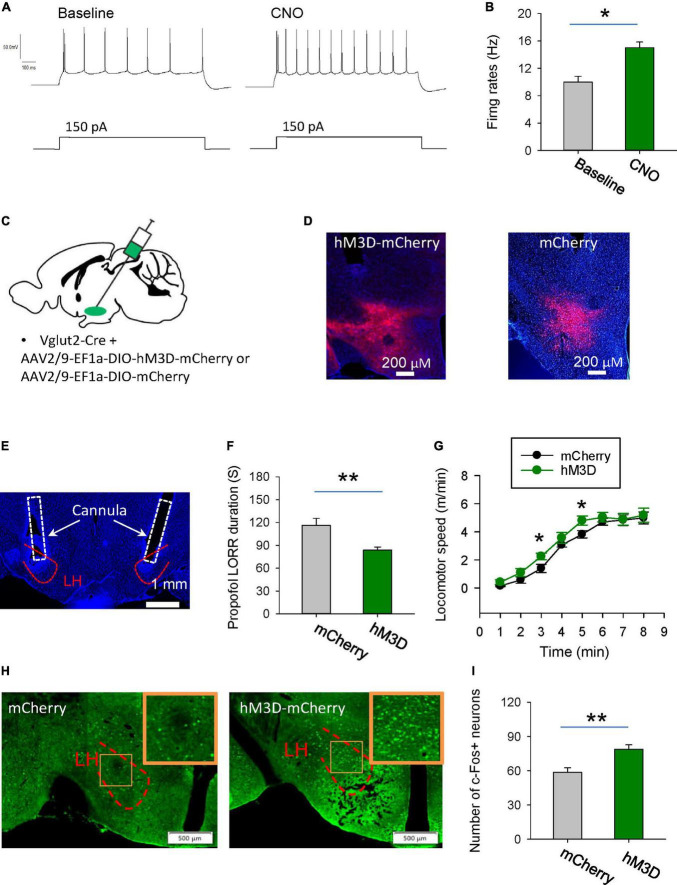
Stimulation of glutamatergic neurons in the LH delays induction of propofol anesthesia. **(A,B)** Effects of CNO on the firing rates of the hM3D-mCherry-positive neurons in the LH. **(C)** Bilateral injection of viruses into the LH region of vglut2-Cre mice. **(D)** Representative images illustrating the expression of the hM3D-mCherry and mCherry in the LH. **(E)** The cannula for application of CNO implanted within the LH. **(F)** Activation of glutamatergic neurons by CNO in the LH decreased the duration of propofol (10 mg/kg, i.v.)-induced loss of LORR. **(G)** Effects of chemogenetic activation of glutamatergic neurons on locomotion of the mice following recovery of the LORR. **(H)** Representative images showing the c-Fos expression in the mCherry and hM3D-mCherry groups. The inset is an enlarged view of c-Fos expression in the LH. **(I)** Effect of chemogenetic activation of glutamatergic neurons on the c-Fos expression in the LH. *,**P < 0.001.

Next, we want to know the behavioral effects of chemogenetic activation of glutamatergic neurons in the LH on the propofol anesthesia. Using whole-cell recordings in the acute brain slices, bath-applied CNO increased the firing rates of hM3D-mCherry-positive glutamatergic neurons, implying that CNO can activate hM3D-mCherry-positive neurons (Figures 4A, B). AAV-DIO-hM3D-mCherry and AAV-DIO-mCherry were restrictedly injected into the LH of vGlut2-Cre mice (Figures 4C, D). At the behavioral level, chemogenetic activation of glutamatergic neurons in the LH increased the duration of propofol-induced LORR (Figures 4E, F). Additionally, the locomotion with CNO injection in the mice with expression of hM3D-mCherry was also enhanced after they recovered from LORR compared with mCherry group (Figure 4G). In particular, there was a 57 and 26% increase in motor speed at 3 min (mCherry: 1.4 m/min; hM3D-mCherry: 2.2 m/min) and 5 min (mCherry: 3.8 m/min, hM3D-mCherry: 4.8 m/min) after recovery of the righting reflex, respectively. These data suggest that activation of the glutamatergic neurons in the LH delay the propofol anesthesia.

The changes in neuronal activity in the LH were also detected by c-Fos staining. After chemogenetic activation of LH glutamatergic neurons during anesthesia, c-Fos positive neurons were significantly increased (Figures 4H, I). These data imply that chemogenetic activation of LH neurons might affect the propofol anesthesia.

## Discussion

Glutamatergic neurons in the LH play an important role in maintenance of wakefulness (Wang et al., 2021). Propofol-anesthetic effects are associated with the glutamatergic neurons in the LH, as chemogenetic inhibition of these neurons increases the duration of propofol-induced LORR and vice versa. These effects of propofol were partially mediated by its inhibitory effects on the glutamatergic neurons in the LH. In sum, these findings may provide novel mechanisms underlying the anesthetic effect of propofol.

In the neocortex and subcortical hippocampus, propofol exerts a direct effect on the cell bodies of excitatory neurons, leading to neuronal hyperpolarization and downregulation of excitability (Xu et al., 2014; Li et al., 2016; Kobayashi and Oi, 2017; Luo et al., 2019). Additionally, propofol can enhance inhibitory inputs, thereby suppressing the activity of these brain regions (Chen et al., 1999; Jeong et al., 2011; Wakita et al., 2013). Consistently, we observed that propofol also inhibits glutamatergic neurons in the LH through both direct and indirect mechanisms. However, further investigation is needed to identify how propofol affects ion channels in the LH. In addition, we mainly used mice to study the effects of propofol. The investigation of propofol’s mechanism of action via the LH in humans necessitates further research.

The wakefulness is regulated by multiple subcortical arousal-promoting systems, such as the acetylcholinergic neurons in the basal forebrain, serotonin neurons in the dorsal raphe, noradrenergic neurons in the locus ceruleus, dopaminergic neurons in the ventral tegmental area and lateral hypothalamus (Brown et al., 2012; Zeitzer, 2013; Venner et al., 2016; Horner and Peever, 2017; Scammell et al., 2017). While the sleep-promoting systems, such as the ventrolateral preoptic nucleus and supraoptic nucleus, inhibit the wakefulness-promoting nuclei and consequently induce the transitions from wakefulness to sleep (Szymusiak et al., 2007; Szymusiak and McGinty, 2008; Brown et al., 2012). It has been reported that anesthetics could inhibit the wakefulness-promoting neurons, including dopamine D1 receptor-positive neurons in the nucleus accumbens, orexinergic neurons in the hypothalamus and medial parabrachial neurons contribute to its anesthetic action (Kelz et al., 2008; Xu et al., 2020; Bao et al., 2021). It should be noted that propofol also inhibited the release of wakefulness-promoting neurotransmitters such as dopamine and acetylcholine during anesthesia (Gamou et al., 2010). Additionally, propofol suppresses the excitability of cholinergic neurons in basal forebrain and noradrenergic neurons in the locus ceruleus (Chen et al., 1999, 2018). The present study demonstrates that propofol inhibits the wakefulness-promoting glutamatergic neurons in the LH effectively and contributes to it anesthetic action. These findings combined with previous research support that wakefulness-promoting systems serve as crucial targets for propofol to induce anesthesia.

The LH contains several types of neurons, forming complex microcircuits. The local neural circuits mainly consist of hypocretinergic neurons-glutamatergic neurons-hypocretinergic neurons, which increases the output of hypocretinergic neurons to promote arousal by positive feedback, and hypocretinergic neurons-GABAergic neurons-melanin-concentrating hormone neurons (Li et al., 2002; Bonnavion et al., 2016). Propofol inhibits glutamate neurons, and may indirectly reduce the activity of hypocretinergic neurons, thereby reducing arousal levels. Of course, the effect of propofol on other neurons has not been reported, and further research is needed in the future. Additionally, the present study did not compare the effects of local injection of saline and CNO in the LH brain region on animal behavior, and the role of local injection of CNO remains to be investigated.

## Materials and methods

### Animal surgery

All mouse care and experimental procedures were approved by the North Sichuan Medical College Guide for the Care and Use of Laboratory Animals. Male adult vGlut2-Cre C57/BL6 mice were used in patch-clamp and behavioral experiments. Vglut2-Cre mice (Stock No: 016963) were obtained from the Jackson Laboratory (USA). All mice were housed in an environment with 12 h light/dark cycle and food and water *ad libitum*. Mice were anesthetized with isoflurane and placed in the stereotaxic apparatus. For pharmacological application of the CNO, a cannula was implanted into separately in the LH region on each side (bregma: AP = −1.50 mm; ML = ± 1.15 mm; DV = −4.7 mm). The cannulas were affixed to the skull by dental cement, and the incision was closed.

For chemogenetic inhibition and activation experiments, Cre-inducible AAV2/9-EF1α-DIO-hM3D-mCherry (BrainVTA Technology Co. Ltd., China) and AAV2/9-EF1α-DIO-hM4D-mCherry (BrainVTA Technology Co. Ltd., China) were bilaterally injected into the LH (bregma: AP = −1.50 mm; ML = ± 1.15 mm; DV = −4.8 mm) of the vGlut2-Cre mice, respectively, with a total volume of about 200 nL. hM4D and hM3D are mutant of G_*i*_-coupled M4 muscarinic receptors and G_*q*_-coupled M3 muscarinic receptors, which are selectively stimulated by the CNO, but not the endogenous acetylcholine, and thus lead to inhibit and activate the neurons, respectively.

### Drug application *in vivo*

The method for drug microinjection was line with the previous study (He et al., 2016). CNO (5 μM, 200 nl) and saline were administrated by an inserted cannula and the syringe pump (Harvard apparatus) with a rate of 100 nl/min. After injection, the inserted cannula was left for additional 2 min to allow for diffusion. Propofol (10 mg/kg, i.v.) was administrated via tail-vein injection. Propofol was injected at 18:00, during which mice spent most time in wakefulness.

### Behavioral tests

Mice were tested for LORR by gently placing them on their backs. Mice were assessed as positive for LORR if they made no obvious attempt to right themselves. During anesthesia, behavioral videos of the animals were recorded. The animals’ movement speed was calculated by employing a customized Matlab script for offline tracking of their body, thereby providing insights into their locomotor activity.

### Histological identification

After completion of the behavioral tests, mice were anesthetized and perfused with saline followed by 4% paraformaldehyde. The brains were placed in 30% sucrose and 4% paraformaldehyde solution for dehydration. Brain sections were prepared and stained with DAPI, and the track of cannula and viral expressions can be identified. The mice with incorrect injection sites were excluded from data analysis. For FISH experiments, brains were sectioned into 14 μm coronal slices, and FISH was carried out by using RNAscope Multiplex Fluorescent Assays V2 (Advanced Cell Diagnostics). The probe Slc17a6 (319171, ACD) was used for detecting vGlut2.

Brains were sectioned into 40 μm coronal slices using a freezing microtome (CM 3050S, Leica). Sections containing the LH were incubated in blocking solution (Beyotime, China) with 1% Triton X-100 at 37°C for 25 min. After that, the sections were incubated in primary antibodies at 4°C overnight. Primary antibody (rabbit anti-c-fos, 1:1000, ab190289, Abcam) were applied. Then, the sections were washed with PBS, transferred into secondary antibody (Alexa Flour 488 donkey anti-rabbit IgG, 1:500, Invitrogen) in PBS and incubated at room temperature for 2 h. Fluorescence images were taken using a microscope digital slide scanner (SlideView VS 200, Olympus). The number of c-Fos positive neurons were counted in the LH region.

### Whole-cell patch clamp recordings

vGlut2-Cre transgenic mice were used in patch camp recordings. Brains were removed after decapitation and placed into solution containing 110 mM NMDG, 110 mM HCl, 2.5 mM KCl, 1.2 mM NaH_2_PO_4_, 25 mM NaHCO_3_, 25 mM Glucose, 10 mM MgSO_4_, 0.5 mM CaCl_2_ (pH 7.2–7.4, saturated with 95% O_2_ and 5% CO_2_). A total 350 μm-thick sections containing LH were cut by an oscillating tissue slicer. Sections were incubated for 15 min at 32°C cutting solution and then transferred to ACSF (120 mM NaCl, 2.5 mM KCl, 1.2 mM NaH_2_PO_4_, 25 mM NaHCO_3_, 25 mM Glucose, 10 mM MgSO_4_, 2 mM CaCl_2_, adjusted to pH 7.2–7.4) saturated with 95% O_2_ and 5% CO_2_ before recording (30°C). Sections were removed to the recording chamber, where the oxygenated ACSF was continuously perfused during the whole-cell recording sessions.

Whole-cell recording was carried out in mCherry-expressing cells of LH. Glutamatergic cells were identified by a microscope equipped with differential contrast optics and an infrared video imaging camera. Recordings were performed with glass pipettes (4–6 MΩ) filled with an solution containing 125 mM potassium gluconate, 20 mM KCl, 10 mM Hepes, 1 mM EGTA, 2 mM MgCl_2_•6H_2_O, 4 mM ATP (pH 7.2–7.4). HEKA EPC-10 amplifier was used for recording of the signals with digitalizing at 10 kHz and filtering at 2 kHz. Data were further acquired and analyzed by using PATCHMASTER and IGOR 5.0 software.

To test the effects of propofol on activity of glutamatergic neurons in the LH, inward currents were injected to depolarize the membrane potential to −55 mV and evoke tonic firing. After recording baseline, propofol was perfused for 1 min and followed with washed out.

### Data analysis

Data were presented as the means ± S.E.M. Student’s *t*-test, one-way repeated-measures’ analysis of variance, two-way repeated-measures’ analysis of variance, and Fisher’s protected least significant difference *post-hoc* testing were conducted for statistical analyses. Significant differences were accepted as *P* < 0.05.

## Data availability statement

The raw data supporting the conclusions of this article will be made available by the authors, without undue reservation.

## Ethics statement

The animal studies were approved by the North Sichuan Medical College Guide for the Care and Use of Laboratory Animals. The studies were conducted in accordance with the local legislation and institutional requirements. Written informed consent was obtained from the owners for the participation of their animals in this study.

## Author contributions

WD: Conceptualization, Funding acquisition, Project administration, Supervision, Writing – original draft, Writing – review and editing. YH: Investigation, Writing – original draft. YX: Investigation, Methodology, Writing – review and editing. LL: Investigation, Writing – review and editing. XF: Investigation, Writing – review and editing. FC: Conceptualization, Supervision, Validation, Writing – original draft, Writing – review and editing.
